# Bridging the knowledge gap: Thai parents’ perspectives on dengue infection and its vaccination and the need for targeted promotion

**DOI:** 10.1371/journal.pntd.0013920

**Published:** 2026-01-20

**Authors:** Donruedee Kamkhoad, Jirarporn Tunksakool, Nopporn Apiwattanakul, Jumpee Granger

**Affiliations:** 1 Ramathbodi School of Nursing, Faculty of Medicine Ramathibodi Hospital, Mahidol University, Bangkok, Thailand; 2 Division of Infectious Disease, Department of Pediatrics, Faculty of Medicine Ramathibodi Hospital, Mahidol University, Bangkok, Thailand; George Washington University Medical Center, UNITED STATES OF AMERICA

## Abstract

**Background:**

Dengue infection is endemic in Thailand, with children being the most impacted group. Various measures, including the dengue vaccine (recently recommended in Thailand since 2017 for Dengvaxia and 2023 for Qdenga), have been implemented to prevent and manage the disease. However, the rate of infection among Thai children is still high. This study explored Thai parents’ knowledge and attitudes toward dengue infection and its preventive vaccine, guided by the 4C Model for vaccine hesitancy.

**Methodology:**

This cross-sectional study employed a questionnaire survey, utilizing five investigator-developed instruments. Survey items were grouped into the components of the 4C Model. Data analysis involved descriptive, Pearson’s correlations, and Binary Logistic Regression statistics.

**Findings:**

Among 400 participating parents in this study, high positive attitudes toward general childhood vaccination (mean score = 41.18/60) and a high level of dengue infection knowledge (mean score = 9.08/12) were observed. Common prevention methods included destroying larval breeding sites and mosquito bite prevention. Most parents viewed dengue infection as a serious disease (mean score = 7.65/10), perceiving impacts like children’s school absenteeism (93%) and parental work time off (80.75%). Regarding government management of dengue, a substantial proportion (~35%) expressed no opinion. Parents exhibited an intermediate level of dengue vaccine knowledge (mean score = 5.06/10), with most unaware of its effectiveness or complex recommendations. Most (63.5%) expressed dengue vaccine acceptance, with 9.75% having vaccinated their children and 59.56% planning to do so. All 4C model components except Calculation were weakly but significantly correlated with parents’ dengue vaccine acceptance, with Complacency being the strongest (r = -.149, p = .05). Only Complacency independently predicted dengue vaccine acceptance among Thai parents (OR=0.897, p = .031).

**Conclusions:**

This study provides insights into Thai parents’ understanding of dengue and its vaccine in children. Findings offer a basis for developing targeted public health campaigns and communication strategies to promote dengue vaccine use in children, and to inform public policy on vaccine affordability.

## Author summary

Dengue infection remains a significant global health challenge, particularly affecting children in endemic regions like Thailand. While vaccines offer a crucial tool for prevention, their impact depends heavily on public acceptance. This study explores Thai parents’ understanding and attitudes towards dengue vaccination, using the established 4C model to identify influencing factors.

We found that despite a general awareness of dengue and vaccine availability, many parents lacked detailed knowledge about the vaccine’s specifics, such as its complex recommendations, effectiveness, or cost. Crucially, a sense of complacency regarding dengue’s threat emerged as the strongest factor influencing vaccine acceptance. Interestingly, even parents working as healthcare providers showed lower vaccination intentions for their own children.

These findings are vital for both scientists and the public, including a countrywide policy for vaccine implementation in the Expanded Program on Immunization (EPI). They illuminate key barriers to vaccine uptake in a real-world setting, suggesting that effective public health campaigns, especially healthcare providers’ advice, must go beyond basic information provision. By understanding specific concerns, addressing financial barriers, and countering complacency, public health initiatives can be better tailored to increase dengue vaccine acceptance, ultimately enhancing disease control and improving child health in affected communities.

## Introduction

Dengue infection is caused by the Dengue virus (DENV), which is transmitted by the Aedes mosquito. There are four DENV serotypes that are antigenically similar yet distinct from one another (DENV1, DENV2, DENV3, and DENV4). Patients with this disease exhibit a range of clinical symptoms classified by the WHO, ranging from mild to severe. These include: a) Undifferentiated fever, which resembles common viral infections and is often found in infants or young children; b) Dengue fever (DF), characterized by body aches and small petechial rashes; c) Dengue hemorrhagic fever (DHF), marked by low platelet counts and a risk of shock (dengue shock); and finally, d) Expanded dengue syndrome, characterized by dengue infection with atypical manifestation including prolonged shock which leads to organ failure, such as liver or kidney failure [1]. Worldwide, as of October 2025, dengue cases surpassed 4.5 million, with over 3,000 associated deaths reported [2]. Currently in Thailand, while dengue infection is increasingly found among adults, children remain the group with the highest reported cases. According to the Department of Disease Control, Thailand, the annual incidence rate of dengue infection among Thai people has steadily risen. It climbed from 17.37 per 100,000 populations in 2022 to 47.68 in 2023, and further to 60.24 in 2024. As of December 17^th^, 2025, children account for the highest incidence, with 27.82% of all patients falling within the 5–14 years age group, followed by 21.82% among those aged 15–24 years [3]. Like in other dengue-endemic areas, multiple strategies have been implemented to control dengue in Thai communities. These include public education, mosquito bite prevention, elimination of Aedes mosquito breeding sites, and the recent introduction of a dengue vaccination program [4].

For more than 40 years since dengue fever was recognized by the WHO South-East Asia Region, research on the development of dengue vaccines for all four DENV serotypes has been conducted in various countries, including Thailand [5]. In 2015, Dengvaxia (CYD-TDV), a live-attenuated dengue vaccine developed by Sanofi Pasteur was licensed for clinical use [6]. Based on evolving effectiveness and safety data, the SAGE Working Group on Dengue Vaccines and WHO Secretariat launched revised recommendations in 2018, advocating for its use in individuals aged 9–45 years living in highly endemic settings, with a strong preference for vaccinating only those with prior laboratory-confirmed dengue infection [7]. Dengvaxia has been recently approved by the US Food and Drug Administration (FDA) for preventing dengue caused by serotypes 1–4 in individuals 6–16 years old who have a confirmed history of dengue infection and live in areas where the disease is common in 2019 [8]. In Thailand, Dengvaxia was approved by the Thai FDA in 2016 for individuals aged 9–45 years [9] and was recommended in 2017 by the Pediatric Infectious Disease Society of Thailand as an alternative (self-paid) vaccine for children [10]. In addition to the complex pre-vaccination screening required for Dengvaxia, controversies surrounding its effectiveness, and safety concerns of vaccination in the seronegative group [11], have led to reduced recommendations from healthcare providers.

Besides Dengvaxia, Qdenga (TAK-003), a vaccine developed by Takeda, has recently received licensing in 2022 from the European Medicines Agency (EMA) and the UK’s Medicines and Healthcare products Regulatory Agency (MHRA) for individuals aged four years and older [12]. The Qdenga vaccine has shown effectiveness against virologically confirmed dengue regardless of serostatus, with higher efficacy against symptomatic dengue in adolescents, and has led to a reduction in hospitalizations [13]. In Thailand, it was officially registered for individuals aged 4 years old and above on May 8^th^, 2023 [14], and recommended by the Pediatric Infectious Disease Society of Thailand as an alternative (self-paid) vaccine for children later in 2023 [15]. Although some government campaigns in Thailand, collaborating with Takeda, have offered free DENV vaccines to children aged 7–10 in currently specific provinces as of June 2025, most families still bear the cost out-of-pocket [16]. Given this financial limitation, and similar to challenges faced in other countries [17], the acceptance of this vaccine by children’s caregivers in Thailand is crucial for further preventing dengue infection and complications among children in Thailand.

Attitudes toward the dengue vaccine, including acceptance and willingness to pay, have been explored in various aspects globally. A recent systematic review by Orellano and colleagues found an overall dengue vaccine acceptance rate of 88.3% among 11,159 individuals, including 2,012 parents, across two high-income and 17 upper- or lower-middle-income countries [17]. Several factors related to dengue vaccine acceptance among the adult population have been reported as attitudes toward vaccination, socioeconomic status, knowledge about dengue infection [18], accessibility to the public, and confidence in the healthcare system and government [19]. Regarding parents’ attitudes toward the dengue vaccine for children, acceptance rates vary widely, from 70.2% to 95.5% [17]. However, the factors influencing parents’ vaccine acceptance have been reported consistently across studies. Parents in the Philippines [20] or Indonesia [21] reported that their personal previous vaccine and dengue experiences played important roles in their accepting the dengue vaccine for their children. Moreover, parents in the Philippines highlighted their trust in public health institutions and the impact of negative messaging regarding the dengue vaccine as other important factors [20]. Other factors, such as the cost of the vaccine, disease prevention practices, and the duration of vaccine protection, also influence the willingness to pay for the vaccine [22].

Dengue vaccines are still in the early stages of widespread implementation in Thailand. To date, only one study has examined attitudes toward the dengue vaccine among the Thai population, focusing solely on adults in Yala province in 2016 [23]. The study reported a 95% acceptance rate and a willingness to pay 4,500 Baht (138 USD) for a three-dose vaccination series. No surveys on vaccine uptake have been conducted, and no studies have specifically explored these attitudes among caregivers of Thai children. To address this critical gap, this study examines attitudes toward dengue infection and vaccination among caregivers of Thai children, including parents, family members, and relatives (hereafter collectively referred to as parents). The findings will provide vital data for developing effective communication strategies and optimizing approaches to promote dengue vaccination among Thai children.

### Objectives

This study aims to:

1. Describe attitude toward dengue vaccination in children among Thai parents.
2. Describe knowledge and attitudes about dengue infection in children among Thai parents.
3. Understand the attitudes of Thai parents toward the current dengue vaccine for children.
4. Examine correlations among relevant factors and Thai parents’ acceptance of the dengue vaccine for their children.

## Methodology

### Ethical consideration

Ethical approval for this study was obtained from the Institutional Review Board of Faculty of Medicine Ramathibodi Hospital, Mahidol University (COA No. MURA2024/771), and the study was conducted in accordance with principles of the Declaration of Helsinki. This study involved only caregivers as participants; accordingly, written informed consent was obtained from all participants prior to their participation. Information regarding children’s demographics was collected solely through caregiver reports to ensure no minors were directly involved in this study. To provide a clear understanding of the study, participants were informed of the study’s purpose, the nature of their involvement, and the estimated time required to complete the survey. Participants were then provided with the survey via a link, QR code, or paper form to complete. They were also assured of the confidentiality of their identities and survey responses, as well as their option to decline participation at any time without consequences.

This descriptive study employed a quantitative design, guided by the 4C model for vaccine hesitancy [24], utilizing investigator-developed questionnaires to explore each study variable.

To conceptualize dengue vaccine hesitancy among Thai parents, this study was guided by the 4C model of vaccine hesitancy, a widely-used framework for understanding the behavioral drivers of vaccine acceptance [24]. The 4C model was an expanded version of the 3C model developed by the Strategic Advisory Group of Experts (SAGE) on Immunization focusing on understanding and addressing vaccine hesitancy [25]. Both models outline different factors that influence individuals’ decisions regarding vaccination, but the 4C model expands upon the 3C model by adding an additional dimension. The four factors of the 4C include:

1. Confidence: Trust in the effectiveness and safety of vaccines, the system that delivers them, and the policymakers’ motivations.
2. Complacency: Perception that the risks of vaccine-preventable diseases are low, and vaccination is not a necessary preventive action.
3. Convenience: Physical availability, affordability, willingness-to-pay, geographical accessibility, and the appeal of immunization services.
4. Calculation: The extent to which individuals engage in extensive information searching and weigh the benefits and risks of vaccination.

This model was chosen in this study due to its comprehensive nature, encompassing factors at both personal and environmental levels, which are crucial for understanding Thai parents’ acceptance of the dengue vaccine for their children.

### Study hypotheses

We hypothesize that higher levels of Confidence, Convenience, and Calculation regarding the dengue vaccine, and lower levels of Complacency, will be positively associated with higher vaccine acceptance among Thai parents.

### Study measures

There are five investigator-developed questionnaires including

1. The Demographic Data Questionnaire for Parents includes their age, gender, religion, educational level, occupation, household income per month, current living area, vaccination history in the past one year, and others.
2. The Demographic Data Questionnaire for Child collects the child’s data from the parent. This includes the child’s date of birth, gender, educational level, underlying diseases, and vaccination history, including the newly introduced COVID-19 vaccine, as its recent introduction could potentially influence parental decision-making regarding dengue vaccine acceptance. This is the information for each child if the parent has more than one child in the household.
3. The Parents’ Attitude Toward Children’s Vaccination Questionnaire consists of 15 items that measure the level of agreement of parents with various statements about children’s vaccination. This approach allows us to evaluate how each related aspect, as mentioned in each statement, influences the parents’ attitudes toward vaccination. The questionnaire is based on previous studies regarding parents’ acceptance of their children’s vaccination [26,27] and the 4C model [24]. Responses are recorded on a 5-point Likert scale, ranging from 0 (strongly disagree) to 4 (strongly agree). For the 10 negative statements (items 5, 6, 7, 8, 10, 11, 12, 13, 14, 15), responses were reversed before summation. The total possible score ranges from 0 to 60. Consequently, higher summary scores indicate a more positive attitude toward children’s vaccination in general. The scale demonstrated good internal consistency, with a Cronbach’s alpha of 0.89.
4. The Parents’ Knowledge and Attitude toward Dengue Infection in Children Questionnaire includes two sections based on previous studies about dengue infection [19,21,28,29], official websites providing information regarding dengue infection in Thailand [1], and the 4C model [24].
  a. Parents’ Knowledge about Dengue Infection in Children
    i. A total of 12 true or false questions regarding general information about dengue infection. The possible scores are 0–12. A higher score indicates higher knowledge of dengue infection in children.
    ii. An item asks about the parents’ strategies for preventing dengue infection in their family. Thirteen response choices are strategies they can select to prevent dengue infection, while one choice is ‘do nothing.’ Selecting ‘do nothing’ may indicate parent complacency or lack of knowledge about dengue infection in children.
    iii. A total of three items asking about self-report of a prior history of dengue infection diagnosis for themselves, their children, and their neighbors to capture the parents’ perceived risk and lived experience with the disease.
  b. Parents’ Attitude toward Dengue Infection in Children
    i. A 10-point scale asking how severe the parent thinks dengue infection is. The possible scores are 0–10. A higher score indicates a higher perceived severity of dengue infection by the parent.
    ii. An item asking about the potential consequences after a child is diagnosed with dengue infection. Parents can select more than one choice among 11 potential health, social, or economic consequences after a child is diagnosed with dengue infection.
    iii. A total of seven items asking how much the parent agrees with each statement about dengue infection in children using a five-point Likert scale, ranging from 0 (strongly disagree) to 4 (strongly agree). For the 4 negative statements (items 2, 3, 4, 5), responses were reversed before summation. The total possible score ranges from 0 to 28. A higher score indicates a greater concern about dengue infection among parents.
5. The Parents’ Knowledge and Attitude toward Dengue Vaccine in Children Questionnaire includes 3 sections based on previous studies about the dengue vaccine [19,21,28,29], an official website providing information regarding the dengue vaccine in Thailand [30], and the 4C model [24].
  a. Parents’ Knowledge about the Dengue Vaccine in Children

A 10-statement section regarding the dengue vaccine with true, false, or “do not know” responses. The possible scores are 0–10. A higher score indicates higher parent knowledge of the dengue vaccine.

b. Parents’ Dengue Vaccine Acceptance

Information on parents’ and children’s dengue vaccination history, as well as vaccination plans for those who have not yet taken their children to be vaccinated.

c. Parents’ Attitude toward the Dengue Vaccine
  i. A total of 12 items asking how much the parent agrees with each statement about the dengue vaccine in children using a five-point Likert scale, ranging from 0 (strongly disagree) to 4 (strongly agree). For the 11 negative statements, all except item 9, responses were reversed before summation. The total possible score ranges from 0 to 48. Consequently, higher summary scores indicate a more positive attitude toward the dengue fever vaccine. The internal consistency reliability of the scale was acceptable, as evidenced by a Cronbach’s alpha coefficient of 0.87.
  ii. An Item asking about the information sources the parent usually uses for health-related information, including vaccines. There are 15 response choices, including an ‘other, please specify’ option.

The study measures were reviewed by three experts, one in children’s infectious diseases and the other two in children health promotion in Thailand, to ensure their content validity and relevance to the Thai healthcare context. The content validity index (CVI) for each questionnaire was acceptable. Specifically, for The Parents’ Attitude Toward Children’s Vaccination Questionnaire, the S-CVI/Ave was 0.95 and the S-CVI/UA was 0.86. The Parents’ Knowledge and Attitude toward Dengue Infection in Children Questionnaire demonstrated perfect validity with S-CVI/Ave and S-CVI/UA both at 1.0. Finally, for the Parents’ Knowledge and Attitude toward Dengue Vaccine in Children Questionnaire, the S-CVI/Ave was 0.94 and the S-CVI/UA was 0.83. Then, these questionnaires were uploaded on REDCap, a secure and user-friendly data management system [31,32], to collect and manage data from study participants which was monitored and maintained by the Faculty of Medicine Ramathibodi Hospital, ensuring robust data security and compliance with institutional standards.

### Study sample

Eligible individuals who were (a) older than 18 years old, (b) the parent or caretaker of children aged 4 to less than 18 years old, (c) able to communicate and understand the Thai language, and (d) currently residing in Thailand.

The calculation utilized the standard formula for sample size determination based on margin of error [33]:


$$\text{n}=\frac{Z^{\text{2}}\times p(\text{1}-p)}{E^{\text{2}}}$$

n = Sample

Z = Confidence Level = 1.96 (95%)

E = Margin of error = 0.05 (5%)

p = Population proportion = 0.5 (50% as it provides the maximum variability)


$$\text{n}=\frac{Z^{\text{2}}\times p(\text{1}-p)}{E^{\text{2}}}=\frac{{\text{1}.\text{9}\text{6}}^{\text{2}}\times \text{0}.\text{5}(\text{1}-\text{0}.\text{5})}{{\text{0}.\text{0}\text{5}}^{\text{2}}}=\text{3}\text{8}\text{5}$$

Of the 563 survey questionnaires initiated, 400 were completed, providing an adequate for the exploratory, descriptive, and correlation analyses planned in this study.

### Data collection

Following study approval, this study employed a combination of online and offline recruitment strategies to reach potential participants. Online recruitment involved disseminating study announcements and flyers on social media (e.g., Facebook). However, within the first two weeks, most participants who completed the online survey were predominantly from Central Thailand or Bangkok. To obtain a broader demographic representation, we subsequently closed the REDCap platform and shifted to a targeted, offline recruitment strategy using six research assistants in different regions. These assistants utilized snowball sampling to further broaden the recruitment of eligible parents across all regions of Thailand. Once participants provided their informed consent, they were given a link to access or paper form to complete the study survey. The survey took approximately 15–20 minutes to complete.

### Data analysis

After data collection was completed, all survey data from both REDCap and paper-based survey were exported to SPSS files for subsequent analysis. This study primarily used descriptive statistics to describe the characteristics of study participants and children as well as parents’ responses from The Parents’ Attitude Toward Children’s Vaccination Questionnaire, The Parents’ Knowledge and Attitude toward Dengue Infection in Children Questionnaire, and The Parents’ Knowledge and Attitude toward Dengue Vaccine in Children Questionnaire.

To explore the relationships among the components of the 4C model and parents’ acceptance of the dengue vaccine, each item from The Parents’ Attitude Toward Children’s Vaccination Questionnaire*,* The Parents’ Knowledge and Attitude Toward Dengue Infection in Children Questionnaire, and The Parents’ Knowledge and Attitude Toward Dengue Vaccine in Children Questionnaire was categorized under a corresponding component of the 4C model. For example, the statement “3.1 It’s necessary for children to receive vaccinations” was assigned to the Confidence component, while the item “3.10 Taking children for vaccination hinders parents’ work” fell under the Convenience component (see S1 Table). Among the 4C components, 36 items were assigned to Confidence, 6 to Convenience, 23 to Complacency, and 1 to Calculation. For each item within the 4C model, favorable responses (e.g., “strongly agree” and “agree” for positive statements like “It’s necessary for children to receive vaccinations” or the correct answer for knowledge items) were assigned a score of 1, while other responses (which includes “do not know,” “disagree,” and “strongly disagree”) received a score of 0. For 23 items representing Complacency, responses were reversed from 1 to 0 before summation. A total score for each 4C component (Confidence, Convenience, Complacency, Calculation) was then calculated by summing the scores of all items assigned to that component (see S1 Table). To explore the relationships between these factors and parental acceptance of the dengue vaccine, Pearson’s correlations were conducted between each of the 4C component scores and participants’ reported dengue vaccine acceptance or their plan to vaccinate their children. Following the correlation analysis, a Binary Logistic Regression was performed to explore the unique contribution of the 4C model components as predictors of Thai parents’ dengue vaccine acceptance for their children.

## Results

A total of 563 questionnaires were initiated, of which 400 were completed (completion rate: 71%). Among these 400 participants residing in all parts of Thailand, the majority were from the Central region, including Bangkok (21.75%), with the fewest from the Western region (12%) (see Table 1). The Thai population is regarded as relatively homogeneous, with only a small proportion belonging to ethnic minority groups. A majority of the participants were female (78.25%) and parents to the child (76.75%), with an average age of 37.86 years (SD = 8.84, range = 19–68 years). Most identified as Buddhist (87.5%), held a Bachelor’s degree (48%), and worked in non-healthcare professions (82.75%). Household income was most frequently reported as more than 45,001 Baht (26.25%), followed by less than 15,000 Baht (24.25%). A total of 562 children were identified from parents’ surveys. The children had a mean age of 10.03 years (SD = 3.96; range = 4.05-17.95), with 53.55% aged 4–10 years and 46.45% aged 10–18 years. Most were Buddhist (85.59%), enrolled in elementary school (49.64%), and had no underlying diseases (87.9%).

**Table 1:** Sample Characteristics (n = 400).

Variable	n(%)
Sex	
Female	313 (78.25)
Male	87 (21.75)
Age (years)	
Means (SD)	37.86 (8.84)
Range	19-68
Religion	
Buddhist	350 (87.5)
Muslim	35 (8.75)
Christian	13 (3.25)
Others	2 (0.5)
Educational level	
Primary school	21 (5.25)
Lower secondary school	35 (8.75)
Upper secondary school	55 (13.75)
Vocational diploma	30 (7.5)
Bachelor’s degree	192 (48)
Graduate Degree	67 (16.75)
Marital status	
Single	93 (23.25)
Married and still together	263 (65.75)
Married but separated	14 (3.5)
Widowed	11 (2.75)
Divorced	19 (4.75)
Occupation	
Healthcare providers	71 (17.75)
Non-healthcare providers	329 (82.75)
Household income (Baht) per month (Means (SD) = 37,635(48,727.64))	
< 15,001 Baht	97 (24.25)
15,001 – 25,000 Baht	92 (23)
25,001 – 35,000 Baht	60 (15)
35,001 – 45,000 Baht	46 (11.5)
> 45,001 Baht	105 (26.25)
Relationship with children	
Parent	307 (76.75)
Grandparent	18 (4.5)
Others	75 (18.75)
Regions of Thailand (current living area)	
Northern	57 (14.25)
Northeastern	66 (16.5)
Western	48 (12)
Eastern	66 (16.5)
Southern	76 (19)
Central including Bangkok	87 (21.75)
Vaccination history in the past year	
Yes	147 (36.75)
No	254 (63.5)
Numbers of children aged 4 to less than 18 years in the family	
1 person	259 (64.75)
2 people	120 (30)
3 people	21 (5.3)
Children’s demographic information (n,children = 562)	
Sex	
Female	272 (48.4)
Male	290 (51.6)
Age (years)	10.03 (3.96)
Means (SD)	4.05-17.95
Range	
4–10 years	301 (53.55)
10–18 years	261 (46.45)
Religion	
Buddhist	481 (85.59)
Muslim	64 (11.39)
Christian	15 (2.67)
Others	2 (0.35)
Educational level	
Have not started the school	16 (2.85)
Nursery/kindergarten	111 (19.75)
Elementary school	279 (49.64)
Middle school	97 (17.26)
High school	59 (10.50)
Underlying disease(s)	
Yes	68 (12.1)
No	494 (87.9)
Children’s vaccination history (n,caregiver = 400)	
Mandatory vaccines per Pediatric Infectious Disease Society of Thailand	
Always attended appointments, completed as scheduled	381 (95.25)
Missed appointments sometimes (n = 19)	19 (4.75)
Continued the vaccines	1 (5.26)
Stopped receiving the vaccines	18 (94.74)
COVID-19 vaccines	
Never received	124 (31)
Received completely	269 (67.25)
Received incompletely	7 (1.75)
Alternative vaccines	
Never received	198 (49.5)
Received	202 (50.5)

Regarding children’s vaccination history, nearly all parents (95.25%) reported consistently attending all recommended appointments for mandatory vaccines, as advised by the Pediatric Infectious Disease Society of Thailand. Among those who reported occasionally missing appointments, they indicated having stopped their children from receiving further vaccines. The most commonly reported reasons included forgetting, schedule conflicts, being too busy, or unawareness of the appointment. A smaller proportion of parents cited a lack of money or no one available to take the child to the hospital. Among the 69% of parents whose children had received COVID-19 vaccines, 1.75% reported incomplete COVID-19 vaccination. Various factors contributed to this, including discontinuation after a prior COVID-19 infection, forgotten appointments by caregivers, a perception that the COVID-19 situation was improving, and fear of the vaccine.

In terms of alternative vaccines for children, about half of all parents (50.5%) reported taking their children to receive at least one. The most common alternative vaccines administered to these children were influenza, followed by the Varicella vaccine and the Invasive Pneumococcal Disease (IPD) vaccine.

### Parents’ attitudes toward children’s vaccination

With a maximum possible score of 60, the average composite score for parents’ attitudes toward children’s vaccination was 41.17 (SD = 9.5, range = 18–60), indicating predominantly positive attitudes toward childhood vaccination in general (see S2 Table). An analysis of specific statements revealed that more than half of all parents reported agreeing or strongly agreeing with positive statements addressing the significance and benefits of vaccination in general for children as well as their trust in the healthcare system. More than half of all parents disagreed or strongly disagreed with statements suggesting that vaccination is troublesome (69.25%), not beneficial (73.75%), disruptive to work (67.5%), or detrimental to a child’s natural immunity or strength (55.25%).

For statements concerning potential risks, such as “Receiving vaccines introduces pathogens into the body, which might cause the child to develop that disease” and “Vaccination might cause disability in children,” most parents reported having no opinion or indicated they did not know. Regarding the financial aspect, a statement like “Taking children for vaccination is expensive” gathered nearly equal responses, with approximately 30% of parents reporting both agreement and disagreement. Conversely, more than half of parents (53%) agreed with the statement “Some children might be allergic to vaccines,” indicating a notable level of awareness or concern about potential allergic reactions.

### Parents’ knowledge regarding dengue infection in children

In terms of parents’ *knowledge* regarding dengue infection in children, the average summary score was 9.08 (SD = 1.39, range = 5–12) out of a possible 12. This suggests that most parents demonstrated a high level of knowledge concerning dengue infection among children (see S3 Table). More than half of all parents provided correct answers to almost every question, with the exception of the item “Aedes mosquitoes mostly bite people during the evening or at night,” which only 45.5% of parents answered correctly.

Regarding *dengue infection prevention*, most parents (99.5%) reported using at least one prevention methods (see S3 Table). Most common dengue infection prevention methods used by Thai parents were eliminating mosquito breeding sites (85%), followed by applying mosquito repellent and/or using mosquito repellent patches (82%) and allowing indoor insecticide spraying when conducted by community leaders (77.5%).

Considering *dengue infection diagnosis history*, only 11.25% of parents reported a personal dengue infection diagnosis, with the majority (88.37%) experiencing it only once (see S3 Table). Regarding their children, 6.25% of parents reported a dengue infection diagnosis, with most (56%) having experienced it only once. Furthermore, 32.5% of parents reported knowing of dengue cases among their neighbors.

### Parents’ attitude toward dengue infection in children

In terms of dengue infection severity perceived by all parents, the average level of perceived severity was 7.65 out of 10 (SD = 2, range 0–10), with 85.68% of parents perceiving high severity (6–10 scores) (see S4 Table).

Regarding possible consequences following dengue infection among children, only one parent (0.25%) reported no perceived consequences. With multiple answers permitted, most parents indicated that dengue infection would lead to their child missing school (93%), followed by the child requiring hospitalization (88.75%), and parents needing to take time off work (80.75%) (see S4 Table).

The average composite score for parents’ attitudes toward dengue infection was 17.95 (SD = 3.67, range = 6–28) out of a possible 28, suggesting that most parents held significant concern about dengue infection in children (see S4 Table). Regarding specific attitudes and perceptions about dengue infection, most parents (95.5%) agreed that it constitutes a major public health problem in Thailand, and over 60% believed it can be both prevented and cured. Almost half of all parents agreed on how the Thai government currently manages dengue infection.

### Parents’ knowledge about dengue vaccine in children

Regarding parents’ knowledge about the dengue vaccine, the average score was 5.06 (SD = 3.27, range = 0–10) out of a possible 10, indicating an intermediate level of knowledge (see S5 Table). Analysis of individual items indicated that while more than half of the parents could correctly identify general facts about the availability and context of the dengue vaccine in Thailand, there remained considerable gaps in understanding specific aspects. Notably, questions pertaining to the vaccine’s type, mechanism of action, effectiveness, side effects, and financial costs were frequently marked as “do not know” by over 50% of participants, indicating significant uncertainty and lack of information in these areas.

### Parents’ attitude toward dengue vaccine in children

In terms of parents’ attitudes toward the dengue vaccine (see S6 Table), the average summary score was 25.75 (SD = 8.5, range = 4–48) out of a possible 48, indicating an intermediate level of positive attitudes.

An analysis of specific statements revealed that nearly half of all parents (48.5%-54.75%) held concerns about the vaccine’s effectiveness and safety. Conversely, 59.25% expressed trust in the healthcare system and medical professionals to administer the dengue vaccine and manage its side effects. While 56.75% believed in building immunity through dengue vaccination, some parents (52.75%-58.25%) deemed the dengue vaccine necessary regardless of a low perceived risk of infection or no neighborhood outbreaks.

Regarding the convenience of taking children to receive the dengue vaccine, more than 60% of parents reported a need to travel far to receive it, while an additional 45.25% believed the vaccine is currently too expensive.

Considering information sources where parents get health-related information including vaccines, all parents selected at least one source. With multiple answers allowed, most parents (86%) received advice from medical professionals, followed by internet search engines (74.5%) and social media (70%) (see S6 Table).

Regarding dengue vaccine acceptance behaviors among Thai parents, only 3.75% of all parents had personally received the vaccine, with 53.33% of this group having completed their vaccination course (see S6 Table). For their children’s dengue vaccination, only 9.75% of all parents (representing 47 children) had taken their children to receive dengue vaccines, and almost half of these 47 children had completed the vaccination. Two parents (representing 3 children) reported discontinuing vaccination before completion due to work-related schedule conflicts. In a sub-analysis of the 71 parents who worked as healthcare providers, 11 had already taken their 13 children to receive the dengue vaccine. Among the 60 parents who had not yet vaccinated their children, a smaller proportion (45%) intended to do so compared to parents who were not healthcare providers (57.14%), although this difference was not statistically significant.

### Correlations among related factors and parents’ acceptance of the dengue vaccine for their children

As shown in Table 2, three of the four 4C components were significantly correlated with dengue vaccine acceptance (p < .05), with Calculation being the exception. The Confidence component (based on 36 items) demonstrated a very weak positive correlation with vaccine acceptance (r = .110, p = .028), as did the Convenience component (6 items) (r = 116, p = .021). In contrast, the Complacency component (23 items) was weakly but negatively correlated with acceptance (r = -.149, p = .003), indicating that greater complacency was associated with lower willingness to vaccinate.

**Table 2:** Correlations among Related Factors and Parents’ Acceptance of the Dengue Vaccine for Their Children (n = 400).

	1.	2.	3.	4.	5.
**1. Dengue vaccine acceptance**	1	.110^a^	.116 ^a^	-.149 ^a^	-.038
**2. Confidence**		1	.355 ^a^	-.315 ^a^	-.076
**3. Convenience**			1	-.294 ^a^	-.060
**4. Complacency**				1	.062
**5. Calculation**					1

^a^ Correlation is significant at the 0.05 level (2-tailed).

A Binary Logistic Regression was performed to examine the unique contributions of 4C components in predicting parental dengue vaccine acceptance (Yes/No). Initial testing of the Binary Logistic Regression model indicated issues with parameter estimation due to the single-item calculation variable, which exhibited properties suggesting quasi-complete separation with the binary outcome. The instability, evidenced by the extreme Standard Error and non-convergent coefficients for this variable, warranted its exclusion. The final reported model therefore includes only the three remaining 4C components: Confidence, Convenience, and Complacency. The full model containing all three predictors was found to be statistically significant, reliably distinguishing between parents who accepted the vaccine and those who did not (Chi-square = 12.265, df = 3, p = .007). The model successfully explained 4.1% of the variance in vaccine acceptance (Nagelkerke R^2^ = .041). Furthermore, the Hosmer-Lemeshow goodness-of-fit test indicated that the model provided a good fit to the data (Chi-square = 3.968, df = 8, p = .860).

**Fig 1:**
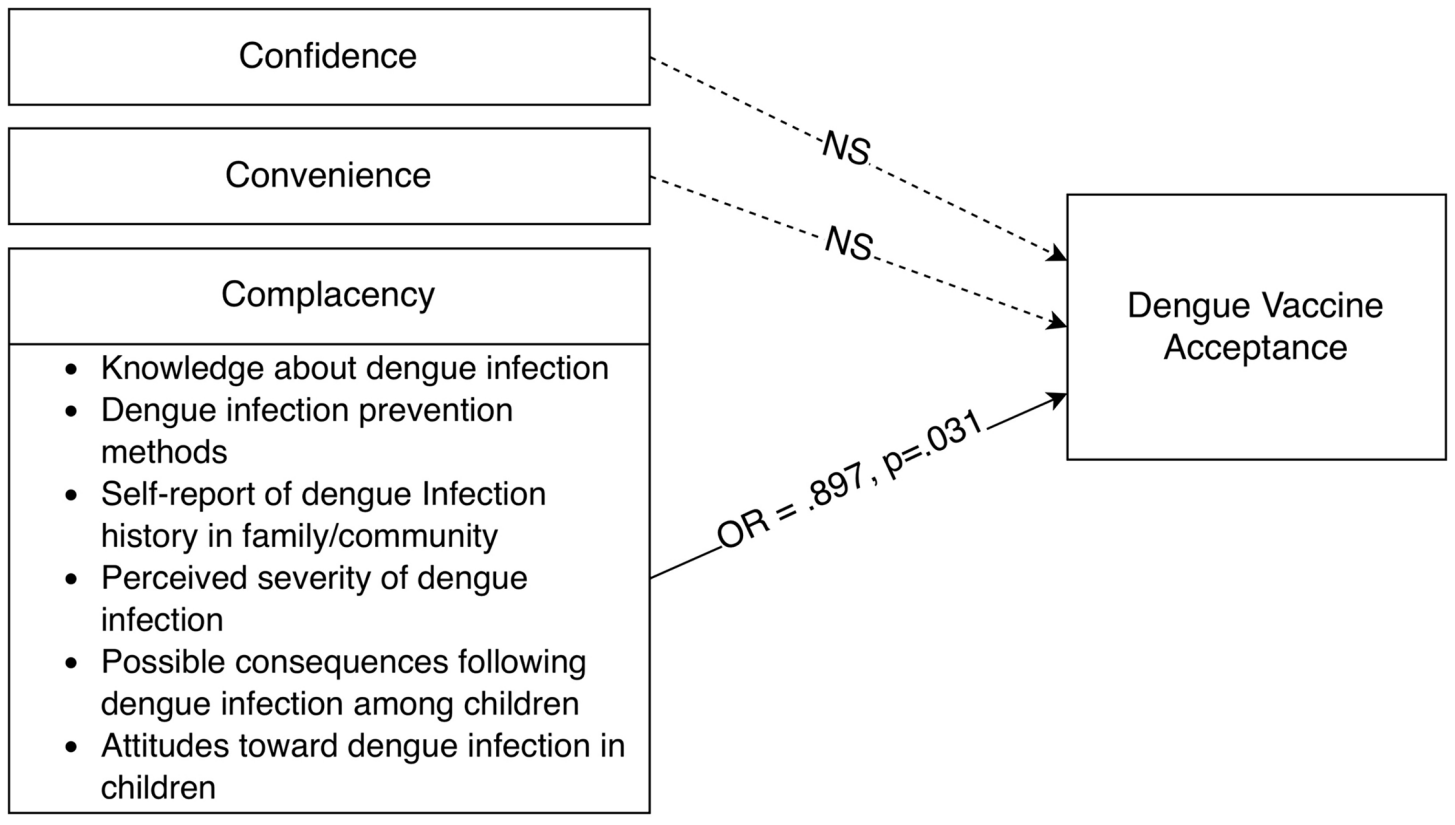
Conceptual Model and Predictive Factors of Dengue Vaccine Acceptance among Thai Parents. Note: The paths represent the results of a Binary Logistic Regression.

Results presented in Table 3 and Fig 1 indicate that only complacency was a statistically significant predictor of dengue vaccine acceptance among Thai parents. The Odds Ratio (OR) for Complacency was 0.897 (p = .031, 95%CI = 0.81-0.99), indicating a statistically significant negative association. Specifically, a one-unit increase in Complacency was associated with a 10.3% decrease in the odds of a parent accepting the dengue vaccine, holding other factors constant. The Confidence (OR=1.021, p = .335) and Convenience (OR=1.090, p = .236) were not statistically significant predictors of vaccine acceptance in this final model.

**Table 3:** Binary Logistic Regression Predicting Parents’ Acceptance of the Dengue Vaccine for Their Children (n = 400).

4C model component	B	S.E.	Wald χ2	df	Sig.	Odds Ratio	95% Confidence Interval
Confidence	.021	.022	.930	1	.338	1.021	[0.98, 1.06]
Convenience	.086	.073	1.403	1	.236	1.090	[0.95, 1.25]
Complacency	-.109	.050	4.647	1	**.031**	.897	[0.81, 0.99]
Constant	.511	.603	.717	1	.397	1.666	

## Discussion

This study describes Thai parents’ attitudes toward vaccination in general, as well as their knowledge of and attitudes toward dengue infection and its preventive vaccines.

Regarding parents’ attitudes toward children’s vaccination in general, we found that most Thai parents held positive attitudes toward children’s vaccination in general, especially mandatory vaccinations, consistent with findings reported by a recent systematic review regarding parental attitudes towards mandatory vaccination [34]. Parents in different countries reported support for mandatory vaccination schemes was reasonably high (73% to 88%). Like parents in Indonesia, most parents in our study viewed the necessity and benefits of vaccination for their children [21]. Several factors within the Thai healthcare system may explain the findings. First, decades of successful national immunization programs have built strong, trusting relationships between healthcare providers and parents. Second, parents trust the consistent, authoritative guidance provided by the Pediatric Infectious Disease Society of Thailand’s annual vaccination schedule announcements [10,15]. Lastly, Thailand’s robust primary healthcare system ensures regular contact between families and healthcare providers, which reinforces positive vaccination messaging. This strong foundation of trust and positive experiences with general vaccination presents an important opportunity for promoting the dengue vaccine among Thai parents. Several possible reasons can be related to Thai healthcare system.

Regarding knowledge of dengue, our findings show that most Thai parents knew some aspects of Aedes mosquitoes as the dengue vector, which is consistent with parents in Grenada who reported knowledge of dengue fever transmission [35]. Moreover, Thai parents demonstrated knowledge about dengue infection among children, especially regarding how children are susceptible to dengue infection and can be infected multiple times. In alignment with this, a study in the Peruvian Amazon identified that parents were aware of how dengue infection could have high impacts on their children due to the inability of young children to articulate their symptoms as a factor making them more vulnerable to complications of this disease [36]. Most Thai parents knew that complications from dengue fever can lead to death. This heightened awareness could be attributed to the widely publicized case in 2016, when a prominent Thai actor (Thrisadee “Por” Sahawawong) died due to complications from dengue infection. This significant event has likely increased public awareness among Thai people since then [37]. However, for the item inquiring whether Aedes mosquitoes mostly bite people during the evening or at night, fewer than half of parents answered correctly. This finding was consistent with a study among parents in the Peruvian Amazon, who similarly believed Aedes mosquitoes bit during both day and night, leading them to emphasize nighttime mosquito bite prevention [36]. Another possible reason could be that dengue prevention messages primarily focus on eliminating breeding sites and using repellents, without detailing the specific mosquito activity times [4]. Consistent with this focus, our findings revealed that most parents primarily eliminated breeding sites, especially by turning over containers that collect still water and used mosquito repellents, similar to practices reported among parents in India [38]. To address this knowledge gap, future communication strategies must be more precise. They should explicitly emphasize preventive measures during the day, such as using repellents during school hours and outdoor activities. Simultaneously, campaigns should clearly differentiate the daytime biting habits of Aedes mosquitoes from other species. This targeted knowledge correction is crucial because a better understanding of when children are most vulnerable to be bitten can significantly improve the effectiveness of prevention efforts and the allocation of public health resources.

Regarding attitudes toward dengue infection, our findings showed that most parents viewed it as a serious illness with high severity. This finding is consistent with studies among parents in other countries [35,36,38]. While parents in the Peruvian Amazon perceived children as the most susceptible group to dengue, which prompted serious dengue control efforts aimed at saving their children’s lives [36], our study found that in addition to concerns about losing their children’s lives, most parents also expressed concerns regarding children missing school and requiring hospitalization following dengue infection. Because the mortality rates of dengue infection in Thailand have been low (around 0.1%) during the past 5 years [3], parents may shift their concerns from mortality to the impact of the disease on children’s daily life. Thai parents have a realistic understanding of dengue’s potential impact on family life. This heightened concern may serve as a cognitive motivator, reinforcing protective behaviors and increasing parents’ openness to vaccine-related information. Consistent with the Health Belief Model [39], which identifies perceived severity as a strong predictor of health-protective actions, these results suggest that interventions that use personal stories from families affected by dengue could be effective in enhancing vaccine uptake. Consequently, parents may be more receptive to vaccination if communications emphasize the practical impact of dengue on daily routines instead of focusing solely on clinical symptoms or mortality statistics.

Regarding the Thai government’s dengue infection management, almost half of all parents generally supported the notion that the government adequately manages dengue infection. However, it is notable that some parents selected “no opinion” or “do not know” for each relevant statement. This phenomenon could be explained by various aspects. For instance, current political conflicts among Thai people might lead individuals not to express definitive opinions on actions performed by the Thai government. Additionally, reports of dengue infection control by the government may not adequately reach Thai population, contributing to their lack of informed responses.

For the knowledge of the dengue vaccine, since the dengue vaccine was first recommended by the Pediatric Infectious Disease Society of Thailand in 2017 for Dengvaxia and in 2023 for Qdenga, our findings showed that Thai parents had an intermediate level of knowledge. Similarly, only 1.48% of parents in the Philippines knew that the dengue vaccine was one of the preventive strategies for dengue fever [40]. From an analysis of specific statements, even though most parents knew that dengue vaccines are currently available in Thailand, they generally lacked detailed knowledge of these vaccines, such as their complex recommendations, side effects, or effectiveness.

Despite the children’s vaccine schedule typically being presented in the Maternal and Child Health Handbook provided by the Bureau of Health Promotion [41], most Thai parents seemed to remain largely uninformed about the dengue vaccine. This could be because the mandatory vaccination schedule for Thai children largely concludes at four years of age, with the next one not occurring until ages 11–12. This gap in routine vaccination, often administered at school, may lead to less frequent use of the handbook and an oversight of alternative vaccines, such as the dengue vaccine for children aged four and older. Furthermore, another possible reason could be that broader educational campaigns prepared by the government specifically on dengue vaccine details might not adequately reach the general Thai population. Being an alternative vaccine, instead of the mandatory one, can also create a discontinuity in information that parents may be unprepared to navigate. The moderate knowledge and attitudes observed in our study may reflect a broader “vaccine fatigue,” amplified by the post-COVID-19 context [42]. Parents might be experiencing information overload and confusion from mixed messages, which is compounded by the availability of two different dengue vaccine brands with varying eligibility criteria. This complexity can lead to uncertainty and delayed decision-making, even among those with generally positive views. Therefore, communication strategies must extend beyond traditional healthcare settings and provide simplified, accurate messaging about the dengue vaccine’s benefits and recommendations.

Considering parents’ attitudes toward the dengue vaccine, our study found that parents generally held an intermediate level of positive attitudes. An analysis of specific statements revealed that while most parents were concerned about the vaccine’s effectiveness, they simultaneously expressed trust in healthcare providers for administering the vaccine and managing its side effects. If health care providers advise and set appointment for the vaccine, the vaccine uptake might increase. We also found that most Thai parents viewed the vaccine as still too expensive. This highlights the significant accessibility challenges that must be addressed to improve the vaccine acceptance.

Despite expressing various concerns regarding the dengue vaccine, a significant proportion of parents had either already vaccinated their children or planned to do so. This finding is consistent with parental intentions reported in other countries [17]. For example, most parents in Indonesia expressed being likely or very likely to vaccinate their children [21].

Interestingly, among parents working as healthcare providers, there appeared to be a lower intention to vaccinate their children compared to non-healthcare parents, although this difference was not statistically significant. This phenomenon is noteworthy and could be attributed to several factors unique to healthcare professionals. They may possess a more nuanced understanding of the dengue vaccine’s complex recommendations and perceived side effects, which can increase the complexity of their decision-making. Additionally, many of these healthcare provider parents may not specialize in fields related to children’s vaccinations or dengue, which could further impact their vaccine uptake. This phenomenon suggests that even knowledgeable individuals, such as healthcare providers, need clear, reassuring messaging that balances facts with emotional impact. As key influencers, their hesitancy could undermine public promotion efforts. Future research should employ qualitative or mixed-methods approaches to fully understand the attitudes and experiences of healthcare providers, as this is essential for developing tailored interventions that boost their own vaccine acceptance and enhance their credibility when communicating with patients.

Regarding the 4C model for dengue vaccine acceptance among Thai parents, our findings supported the model by showing that Confidence, Convenience, and Complacency had a weak but significant correlation with dengue vaccine acceptance (p < 0.05) [24], suggesting that the linear association between these components is not strong. This warrants caution in interpretation; consequently, the subsequent multivariate analysis was essential and showed that only Complacency independently predicted Thai parents’ dengue vaccine acceptance.

Specifically, the Confidence component indicated that parents holding a positive attitude toward general vaccination tended to accept the dengue vaccine. This is consistent with findings from Indonesia, where a supportive attitude towards vaccination practices in general was the most significant factor linked to greater support for dengue vaccination [21].

For the Convenience component, factors such as the self-payment for the dengue vaccine were related to parental acceptance. This aligns with observations in the Philippines, where parents were willing to vaccinate their children if the government offered the vaccine for free [40].

The Complacency component showed that a lower perceived severity of dengue was negatively associated with dengue vaccine acceptance. This means that parents who were more complacent were less likely to accept the vaccine, consistent with findings among parents in the Philippines [20].

The Calculation component, which was measured by only one survey item assessing parents’ general healthcare information sources, was not significantly associated with dengue vaccine acceptance. This lack of association might be because the question was not specific to dengue vaccine, potentially leading to an inaccurate measurement of this component. In contrast to our study where most parents reported obtaining health-related information from healthcare providers, most parents in India relied on television as their primary source of information about dengue [43].

Our findings revealed that among 4C components assessed, Complacency was the only statistically significant predictor of dengue vaccine acceptance among Thai parents. Higher levels of complacency were significantly associated with lower odds of acceptance, indicating it holds the strongest explanatory power in this specific model. This contrasts with a recent narrative review’s finding, which indicated that Confidence emerged as the most influential factor in vaccine hesitancy including dengue vaccine across various populations, followed by Complacency [44]. Unlike novel diseases, such as COVID-19 where Confidence dominates, the long-standing familiarity and relatively low mortality rate of dengue in Thailand shift the primary hesitancy barriers to Complacency (low perceived necessity due to manageable risk) and Convenience (logistical/cost barriers).

Based on these findings, there are several key implications for dengue vaccination programs in Thailand. First, educational campaigns need to specifically target knowledge gaps, such as mosquito biting patterns and detailed vaccine information, including an appointment set for the vaccine. Second, increasing vaccine uptake requires optimizing accessibility and convenience within the existing Thailand’s EPI. This means addressing barriers, such as perceived or actual costs and ensuring adequate availability. Finally, programs should leverage the strong trust in healthcare providers while also addressing their specific concerns to improve overall acceptance.

### Limitations

Some limitations of this study warrant consideration.

First, as the “Calculation” component was assessed using only a single survey item, its operationalization may have been insufficient to fully capture the complexity of parents’ information-seeking and decision-making processes, potentially affecting its construct validity and contributing to its lack of significant association with dengue vaccine acceptance.

Second, our study’s reliance on self-reported data presents a risk of desirability bias. Specifically, responses to subjective questions, such as those concerning attitudes toward dengue management by the Thai government or reported data sources for health information, may have been influenced by a desire to align with perceived community standards or government expectations, potentially impacting the accuracy of the findings.

Third, while valuable insights were gained from the subgroup analysis of parents working as healthcare providers, this specific analysis was not an original aim of the study design. Consequently, the sampling strategy was not specifically tailored to ensure a representative sample of this particular professional group. Future research could benefit from a dedicated study focusing on the knowledge, attitudes, and acceptance of dengue vaccines specifically among Thai healthcare providers to gain more targeted and in-depth understanding.

### Strengths

A significant strength of this study is its comprehensive data collection across all regions of Thailand. This broad geographical representation enhances the external validity and generalizability of the findings, providing a robust and nationally representative understanding of parental knowledge, attitudes, and acceptance of dengue vaccines within the Thai context.

## Conclusion

This study revealed significant knowledge gaps among Thai parents regarding specific aspects of dengue and its vaccine, while attitudes towards the vaccine were generally intermediate but nuanced by concerns over cost and effectiveness. The 4C model provided a valuable framework, with Complacency emerging as the predictor of vaccine acceptance. These findings underscore the need for targeted public health campaigns that not only promote dengue vaccination as a key preventive measure but also strategically address knowledge gaps, enhance communication strategies, and improve vaccine affordability.

## Supporting information

S1 Table Mapping of Survey Items to 4C Model Components. (DOCX)

S2 Table Attitudes toward Childhood Vaccinations (n = 400). (DOCX)

S3 Table Knowledge about Dengue Infection (n = 400). (DOCX)

S4 Table Attitudes toward Dengue Infection in Children (n = 400). (DOCX)

S5 Table Parent’s Knowledge about Dengue Infection Vaccine in Children (n = 400). (DOCX)

S6 Table Parent’s Attitude toward Dengue Vaccine in Children (n = 400). (DOCX)

S1 Data The Anonymized Individual Survey Responses. (XLSX)
