# Supplementary material for: Bridging the knowledge gap: Thai parents’ perspectives on dengue infection and its vaccination and the need for targeted promotion
Source: PLoS Negl Trop Dis. 2026 Jan 20;20(1):e0013920. doi: 10.1371/journal.pntd.0013920 (PMC12829955; doi:10.1371/journal.pntd.0013920)
Supplement: S1 Table — (DOCX) [file pntd.0013920.s001.docx]

**S1 Table.** **Mapping of Survey Items to 4C Model Components**

Survey questionnaires

1. Caregivers’ demographic data and vaccination history
2. Children’s demographic and vaccination history
3. Attitudes towards childhood vaccinations
4. Dengue infection in children (4.1 knowledge about dengue infection, 4.2 Dengue infection prevention methods, 4.3 History of Dengue Infection in the Family, 4.4 History of Dengue Infection in Community, 4.5 perceived severity of dengue infection, 4.6 Possible consequences following dengue infection among children, 4.7 Attitudes Toward Dengue Infection in Children in Thailand)
5. Dengue vaccine (5.1 knowledge, 5.2 dengue vaccine acceptance behavior/intention, 5.3 attitudes toward dengue vaccine, 5.4 Health Information Sources)

| **No.** | **Survey item** | **favorable response** |
| --- | --- | --- |
|  | **Confidence (36 items)** |  |
| 1 | 1.1 caregivers’ vaccination history in the past year | Yes |
| 2 | 2.1 children’s mandatory vaccination history | at least one child completed as scheduled |
| 3 | 2.2 children’s COVID vaccine history | at least one child completed |
| 4 | 3.1 It's necessary for children to receive vaccinations. | A^a^,SA^a^ |
| 5 | 3.2 It's a good idea for children to receive vaccinations. | A^a^,SA^a^ |
| 6 | 3.3 Vaccination benefits children. | A^a^,SA^a^ |
| 7 | 3.4 Vaccination boosts children's immunity. | A^a^,SA^a^ |
| 8 | 3.5 Receiving vaccines introduces pathogens into the body, which might cause the child to develop that disease. | D^a^,SD^a^ |
| 9 | 3.6 Vaccination might cause disability in children. | D^a^,SD^a^ |
| 10 | 3.8 Taking children for vaccination is not worth the benefits received. | D^a^,SD^a^ |
| 11 | 3.9 Healthcare staff should always provide advice on post-vaccination care. | A^a^,SA^a^ |
| 12 | 3.12 Receiving vaccines might have dangerous side effects. | D^a^,SD^a^ |
| 13 | 3.13 Some children might be allergic to vaccines. | D^a^,SD^a^ |
| 14 | 3.14 Children are born with enough natural immunity and don't need vaccines. | D^a^,SD^a^ |
| 15 | 3.15 Receiving vaccines makes children weaker. | D^a^,SD^a^ |
| 16 | 4.7.3 Dengue infection cannot be prevented. | D^a^,SD^a^ |
| 17 | 4.7.6. The government has responded appropriately to dengue infection. | A^a^,SA^a^ |
| 18 | 4.7.7 The government is well-prepared to combat dengue infection. | A^a^,SA^a^ |
| 19 | 5.1.1 Currently, a dengue vaccine is available in Thailand. | True |
| 20 | 5.1.2 Currently, more than one type of dengue vaccine is used in Thailand. | True |
| 21 | 5.1.3 The currently used dengue vaccines are live-attenuated vaccines. | True |
| 22 | 5.1.4 Dengue vaccines require more than one dose. | True |
| 23 | 5.1.5 Children aged 4 years and older can receive the dengue vaccine. | True |
| 24 | 5.1.6 Some dengue vaccines require children to have had dengue infection before vaccination. | True |
| 25 | 5.1.7 Some dengue vaccines may require a blood test before administration. | True |
| 26 | 5.1.8 The effectiveness of dengue vaccines in reducing hospitalization ranges from approximately 60% to 90%. | True |
| 27 | 5.1.9 Side effects of dengue vaccines may include pain at the injection site, mild fever, or skin rash. | True |
| 28 | 5.1.10 Currently in Thailand, individuals must pay for the dengue vaccine out of pocket. | True |
| 29 | 5.3.1 You are concerned about the effectiveness of the dengue vaccine. | D^a^,SD^a^ |
| 30 | 5.3.2 You are concerned about the safety of the dengue vaccine. | D^a^,SD^a^ |
| 31 | 5.3.3 You are concerned about the side effects of the dengue vaccine. | D^a^,SD^a^ |
| 32 | 5.3.4 You are worried that vaccination introduces the dengue virus into the body. | D^a^,SD^a^ |
| 33 | 5.3.5 You will wait until you are sure the dengue vaccine is completely safe before allowing vaccination. | D^a^,SD^a^ |
| 34 | 5.3.6 You believe that because children have weak immune systems, the dengue vaccine may be more harmful than beneficial. | D^a^,SD^a^ |
| 35 | 5.3.7 You do not believe in building immunity through vaccination against dengue. | D^a^,SD^a^ |
| 36 | 5.3.9 You trust the healthcare system and medical professionals to administer the dengue vaccine and manage its side effects. | A^a^,SA^a^ |
|  | **Convenience (6 items)** |  |
| 1 | 2.3 children’s alternative vaccination history | received at least one vaccine |
| 2 | 3.7 Taking children for vaccination is troublesome. | D^a^,SD^a^ |
| 3 | 3.10 Taking children for vaccination hinders parents' work. | D^a^,SD^a^ |
| 4 | 3.11 Taking children for vaccination is expensive. | D^a^,SD^a^ |
| 5 | 5.3.8 You think you would have to travel far to access the dengue vaccine. | D^a^,SD^a^ |
| 6 | 5.3.10 You believe the dengue vaccine is currently too expensive, but would vaccinate your child if the price were lower. | D^a^,SD^a^ |
|  | **Complacency (22 items)** |  |
| 1 | 4.1.1 Aedes mosquitoes are vectors of dengue infection. | True |
| 2 | 4.1.2 Aedes mosquitoes mostly bite people during the evening or at night. | False |
| 3 | 4.1.3 Aedes mosquitoes mostly lay eggs in turbid (cloudy) water. | False |
| 4 | 4.1.4 Dengue infection has a total of four types (serotypes). | True |
| 5 | 4.1.5 Dengue infection is a communicable disease. | True |
| 6 | 4.1.6 All children are susceptible to dengue infection. | True |
| 7 | 4.1.7 Children can get dengue infection multiple times. | True |
| 8 | 4.1.8 Dengue infection tends to becomes more severe upon second infection. | True |
| 9 | 4.1.9 Symptoms of dengue infection in children can be seen with the naked eye. | True |
| 10 | 4.1.10 Children will go into shock every time they have dengue infection. | False |
| 11 | 4.1.11 Some people contract dengue infection but show no symptoms. | True |
| 12 | 4.1.12 Complications of dengue infection can lead to death in children. | True |
| 13 | 4.2 Dengue infection prevention methods | did at least one method |
| 14 | 4.3 History of Dengue Infection in the Family | aware |
| 15 | 4.4 History of Dengue Infection in Community | aware |
| 16 | 4.5 Perceived severity of dengue infection | high perceived severity (6-10) |
| 17 | 4.6. Perceived possible consequences following dengue infection among children | selected at least one |
| 18 | 4.7.1 Dengue infection is a major public health problem in Thailand. | A^a^,SA^a^ |
| 19 | 4.7.2 Dengue infection cannot be cured. | D^a^,SD^a^ |
| 20 | 4.7.4 The threat of dengue infection has been exaggerated by the media. | D^a^,SD^a^ |
| 21 | 4.7.5 The threat of dengue infection has been exaggerated by the government. | D^a^,SD^a^ |
| 22 | 5.3.11 You think that if the risk of contracting dengue is low, vaccination is unnecessary. | D^a^,SD^a^ |
| 23 | 5.3.12 You think that if there is no dengue outbreak nearby, vaccination is unnecessary. | D^a^,SD^a^ |
|  | **Calculation (1 item)** |  |
| 1 | 5.4 Health Information Sources | selected at least one source |
|  | **Outcome – vaccine acceptance (1 item)** |  |
| 1 | 5.2 Dengue vaccine acceptance behavior/intention | have taken or plan to take the children to get dengue vaccine. |

^a^SD= strongly disagree, D=disagree, NO=no opinion/do not know, A=agree, SA=strongly agree
