# Supplementary material for: Bridging the knowledge gap: Thai parents’ perspectives on dengue infection and its vaccination and the need for targeted promotion
Source: PLoS Negl Trop Dis. 2026 Jan 20;20(1):e0013920. doi: 10.1371/journal.pntd.0013920 (PMC12829955; doi:10.1371/journal.pntd.0013920)
Supplement: S2 Table — (DOCX) [file pntd.0013920.s002.docx]

**S2 Table. Attitudes toward Childhood Vaccinations (n=400)**

| **Statement** | **SD^a^**  **n(%)** | **D^a^**  **n(%)** | **NO^a^**  **n(%)** | **A^a^**  **n(%)** | **SA^a^**  **n(%)** |
| --- | --- | --- | --- | --- | --- |
| **1. It's necessary for children to receive vaccinations.** | 1  (0.25) |  | 10  (2.5) | 94 (23.5) | 295 (73.75) |
| **2. It's a good idea for children to receive vaccinations.** |  |  | 10  (2.5) | 118 (29.5) | 272 (68) |
| **3. Vaccination benefits children.** |  | 1  (0.25) | 19 (4.75) | 112 (28) | 268 (67) |
| **4. Vaccination boosts children's immunity.** |  |  | 18  (45) | 109 (27.25) | 273 (68.25) |
| **5. Receiving vaccines introduces pathogens into the body, which might cause the child to develop that disease.** | 77 (19.25) | 110 (27.5) | 102 (25.5) | 56  (14) | 55 (13.75) |
| **6. Vaccination might cause disability in children.** | 83 (20.75) | 114 (28.5) | 127 (31.75) | 38  (9.5) | 38  (9.5) |
| **7. Taking children for vaccination is troublesome.** | 108 (27) | 169 (42.25) | 37 (9.25) | 48  (12) | 38  (9.5) |
| **8. Taking children for vaccination is not worth the benefits received.** | 138 (34.5) | 157 (39.25) | 31 (7.75) | 30  (7.5) | 44  (11) |
| **9. Healthcare staff should always provide advice on post-vaccination care.** | 2  (0.5) | 3  (0.75) | 8  (2) | 104 (26) | 283 (70.75) |
| **10. Taking children for vaccination hinders parents' work.** | 104 (26) | 166 (41.5) | 41 (10.25) | 56  (14) | 33 (8.25) |
| **11. Taking children for vaccination is expensive.** | 35 (8.75) | 123 (30.75) | 62 (15.5) | 122 (30.5) | 58 (14.5) |
| **12. Receiving vaccines might have dangerous side effects.** | 36  (9) | 104 (26) | 134 (33.5) | 91 (22.75) | 35 (8.75) |
| **13. Some children might be allergic to vaccines.** | 9  (2.25) | 22  (5.5) | 79 (19.75) | 215 (53.75) | 75 (18.75) |
| **14. Children are born with enough natural immunity and don't need vaccines.** | 86 (21.5) | 157 (39.25) | 74 (18.5) | 46 (11.5) | 37 (9.25) |
| **15.Receiving vaccines makes children weaker.** | 83 (20.75) | 138 (34.5) | 117 (29.25) | 33 (8.25) | 29 (7.25) |
| **Total score (possible range = 0-60)**  **Means (SD)**  **Range** | **41.17 (9.5)**  **18-60** | | | | |

^a^SD= strongly disagree, D=disagree, NO=no opinion/do not know, A=agree, SA=strongly agree
