# Supplementary material for: Bridging the knowledge gap: Thai parents’ perspectives on dengue infection and its vaccination and the need for targeted promotion
Source: PLoS Negl Trop Dis. 2026 Jan 20;20(1):e0013920. doi: 10.1371/journal.pntd.0013920 (PMC12829955; doi:10.1371/journal.pntd.0013920)
Supplement: S3 Table — (DOCX) [file pntd.0013920.s003.docx]

**S3 Table. Knowledge about Dengue Infection (n=400)**

| **Knowledge about dengue infection** | **Sum score**  **Means (SD)**  **Range** |
| --- | --- |
|  | 9.08 (1.39)  5-12 |
| Statement | **Number of people who answered correctly (%)** |
| 1. Aedes mosquitoes are vectors of dengue infection. | 400 (100) |
| 2. Aedes mosquitoes mostly bite people during the evening or at night. | 182 (45.5) |
| 3. Aedes mosquitoes mostly lay eggs in turbid (cloudy) water. | 218 (54.5) |
| 4. Dengue infection has a total of four types (serotypes). | 338 (84.5) |
| 5. Dengue infection is a communicable disease. | 204 (51) |
| 6. All children are susceptible to dengue infection. | 382 (95.5) |
| 7. Children can get dengue infection multiple times. | 375 (93.75) |
| 8. Dengue infection tends to becomes more severe upon second infection. | 311 (77.75) |
| 9. Symptoms of dengue infection in children can be seen with the naked eye. | 251 (62.75) |
| 10. Children will go into shock every time they have dengue infection. | 262 (65.5) |
| 11. Some people contract dengue infection but show no symptoms. | 315 (78.75) |
| 12. Complications of dengue infection can lead to death in children. | 392 (98) |
| **Dengue infection prevention methods** (multiple answers allowed) | **n (%)** |
| Do nothing | 2 (0.5) |
| Turn over containers that collect still water | 340 (85) |
| Apply mosquito repellent and/or use mosquito repellent patches | 328 (82) |
| Allow indoor insecticide spraying when conducted by community leaders | 310 (77.5) |
| Keep water containers tightly covered | 309 (77.25) |
| Regularly check water storage tanks at home | 292 (73) |
| Kill mosquitoes by hand or with an electric mosquito swatter | 278 (69.5) |
| Regularly ensure that the home’s drainage system is not blocked and doesn’t hold standing water | 277 (69.25) |
| Add mosquito larvae killers (e.g., abate sand or salt) to water containers at home (e.g., vases) | 272 (68) |
| Dispose of garbage that may hold water in covered bins | 262 (65.5) |
| Use mosquito nets | 259 (64.75) |
| Spray insecticide in the house by yourself | 232 (58) |
| Wear long-sleeved shirts and/or long pants | 216 (54) |
| **History of Dengue Infection in the Family or Community** | **n(%)** |
| Have you ever had dengue infection?  - Never  - Not sure  - Yes:  1 time  > 1 time | 269 (67.25)  45 (21.5)  86 (11.25)  76 (88.37)  10 (11.63) |
| Has any child in your household ever had dengue infection?  - Never  - Not sure  - Yes at least one child: (n=25)  1 time  > 1 time | 362 (90.5)  13 (3.25)  25 (6.25)  14 (56)  11 (44) |
| Has anyone you know who lives nearby ever had dengue infection?  - Never  - Not sure  - Yes | 190 (47.5)  80 (20)  130 (32.5) |
