# Supplementary material for: Bridging the knowledge gap: Thai parents’ perspectives on dengue infection and its vaccination and the need for targeted promotion
Source: PLoS Negl Trop Dis. 2026 Jan 20;20(1):e0013920. doi: 10.1371/journal.pntd.0013920 (PMC12829955; doi:10.1371/journal.pntd.0013920)
Supplement: S4 Table — (DOCX) [file pntd.0013920.s004.docx]

**S4 Table. Attitudes toward Dengue Infection in Children (n=400)**

| **The perceived severity of dengue infection in children** | | | | | **n(%)** | |
| --- | --- | --- | --- | --- | --- | --- |
| Means (SD)  Range | | | | | 7.65 (2)  0-10 | |
| - Low perceived severity (0-5)  - High perceived severity (6-10) | | | | | 57 (14.32)  341 (85.68) | |
| **Possible consequences following dengue infection among children (multiple answers allowed)** | | | | | **n(%)** | |
| No perceived consequences | | | | | 1 (0.25) | |
| The child misses school. | | | | | 372 (93) | |
| You have to take time off work. | | | | | 323 (80.75) | |
| You may need to take the child to a clinic or hospital for a check-up. | | | | | 291 (72.75) | |
| The child may need to be hospitalized. | | | | | 355 (88.75) | |
| Unexpected costs arise from hospitalization. | | | | | 264 (66) | |
| You lose income because you have to take time off work to care for the child. | | | | | 205 (51.25) | |
| The child’s underlying health conditions worsen, or there are long-term side effects. | | | | | 225 (56.25) | |
| The child is at risk of getting dengue infection again. | | | | | 260 (65) | |
| If reinfected, the severity of dengue infection will increase. | | | | | 260 (65) | |
| The child’s quality of life worsens. | | | | | 195 (48.75) | |
| The child may die. | | | | | 288 (72) | |
| **Attitudes toward Dengue Infection in Thailand** | | | | | | |
| **Statement** | **SD^a^**  **n(%)** | **D^a^**  **n(%)** | **NO^a^**  **n(%)** | **A^a^**  **n(%)** | | **SA^a^**  **n(%)** |
| 1. Dengue infection is a major public health problem in Thailand. |  | 4  (1) | 14 (3.5) | 193  (48.25) | | 189 (47.25) |
| 2. Dengue infection cannot be cured. | 101 (25.25) | 169 (42.25) | 51 (12.75) | 44  (11) | | 35 (8.75) |
| 3. Dengue infection cannot be prevented. | 129 (32.25) | 164 (41) | 20  (5) | 42 (10.5) | | 45 (11.25) |
| 4. The threat of dengue infection has been exaggerated by the media. | 46 (11.5) | 124 (31) | 143 (35.75) | 53 (13.25) | | 34 (8.5) |
| 5. The threat of dengue infection has been exaggerated by the government. | 41 (10.25) | 129 (32.25) | 148 (37) | 51 (12.75) | | 31 (7.75) |
| 6. The government has responded appropriately to dengue infection. | 22 (5.5) | 57 (14.25) | 155 (38.75) | 121 (30.25) | | 45 (11.25) |
| 7. The government is well-prepared to combat dengue infection. | 19 (4.75) | 42 (10.5) | 150 (37.5) | 137 (34.25) | | 52  (13) |
| **Total score** (possible score = 0-28)  Means (SD)  Range | 17.95 (3.67)  6-28 | | | | | |

^a^SD= strongly disagree, D=disagree, NO=no opinion/do not know, A=agree, SA=strongly agree
