# Supplementary material for: Bridging the knowledge gap: Thai parents’ perspectives on dengue infection and its vaccination and the need for targeted promotion
Source: PLoS Negl Trop Dis. 2026 Jan 20;20(1):e0013920. doi: 10.1371/journal.pntd.0013920 (PMC12829955; doi:10.1371/journal.pntd.0013920)
Supplement: S5 Table — (DOCX) [file pntd.0013920.s005.docx]

**S5 Table. Parent’s Knowledge about Dengue Infection Vaccine in Children (n=400)**

| **Statement** | **Number of people who answered correctly (%)** | **Number of people who chose “do not know” (%)** |
| --- | --- | --- |
| 1. Currently, a dengue vaccine is available in Thailand. | 288 (72) | 98 (24.5) |
| 2. Currently, more than one type of dengue vaccine is used in Thailand. | 201 (50.25) | 179 (44.75) |
| 3. The currently used dengue vaccines are live-attenuated vaccines. | 167 (41.75) | 208 (52) |
| 4. Dengue vaccines require more than one dose. | 188 (47) | 189 (47.25) |
| 5. Children aged 4 years and older can receive the dengue vaccine. | 234 (58.5) | 155 (38.75) |
| 6. Some dengue vaccines require children to have had dengue infection before vaccination. | 107 (26.75) | 197 (49.25) |
| 7. Some dengue vaccines may require a blood test before administration. | 161 (40.25) | 194 (48.5) |
| 8. The effectiveness of dengue vaccines in reducing hospitalization ranges from approximately 60% to 90%. | 138 (34.5) | 247 (61.75) |
| 9. Side effects of dengue vaccines may include pain at the injection site, mild fever, or skin rash. | 160 (40) | 222 (55.5) |
| 10. Currently in Thailand, individuals must pay for the dengue vaccine out of pocket. | 145 (36.25) | 219 (54.75) |
| **Total score** (possible score=0-10)  Means (SD)  Range | 5.06 (3.27)  0-10 | |
