# Supplementary material for: Bridging the knowledge gap: Thai parents’ perspectives on dengue infection and its vaccination and the need for targeted promotion
Source: PLoS Negl Trop Dis. 2026 Jan 20;20(1):e0013920. doi: 10.1371/journal.pntd.0013920 (PMC12829955; doi:10.1371/journal.pntd.0013920)
Supplement: S6 Table — (DOCX) [file pntd.0013920.s006.docx]

**S6 Table. Parent’s Attitude toward Dengue Vaccine in Children (n=400)**

| **Attitude toward Dengue Vaccine in Children** | | | | | | |
| --- | --- | --- | --- | --- | --- | --- |
| **Statement** | **SD^a^**  **n(%)** | **D^a^**  **n(%)** | **NO^a^**  **n(%)** | **A^a^**  **n(%)** | | **SA^a^**  **n(%)** |
| 1. You are concerned about the effectiveness of the dengue vaccine. | 38 (9.5) | 86 (21.5) | 82 (20.5) | 177 (44.25) | | 17 (4.25) |
| 2. You are concerned about the safety of the dengue vaccine. | 41 (10.25) | 85 (21.25) | 72  (18) | 187 (46.75) | | 15 (3.75) |
| 3. You are concerned about the side effects of the dengue vaccine. | 39 (9.75) | 84  (21) | 58 (14.5) | 198 (49.5) | | 21 (5.25) |
| 4. You are worried that vaccination introduces the dengue virus into the body. | 53 (13.25) | 112 (28) | 84  (21) | 133 (33.25) | | 18 (4.5) |
| 5. You will wait until you are sure the dengue vaccine is completely safe before allowing vaccination. | 43 (10.75) | 70 (17.5) | 70 (17.5) | 170 (42.5) | | 47 (11.75) |
| 6. You believe that because children have weak immune systems, the dengue vaccine may be more harmful than beneficial. | 53 (13.25) | 88  (22) | 138 (34.5) | 100 (25) | | 21 (5.25) |
| 7.You do not believe in building immunity through vaccination against dengue. | 66 (16.5) | 161 (40.25) | 110 (27.5) | 53 (13.25) | | 10 (2.5) |
| 8. You think you would have to travel far to access the dengue vaccine. | 68  (17) | 177 (44.25) | 64  (16) | 78 (19.5) | | 13 (3.25) |
| 9. You trust the healthcare system and medical professionals to administer the dengue vaccine and manage its side effects. | 32  (8) | 47 (11.75) | 84  (21) | 196 (49) | | 41 (10.25) |
| 10. You believe the dengue vaccine is currently too expensive, but would vaccinate your child if the price were lower. | 39 (9.75) | 61 (15.25) | 115 (28.75) | 140 (35) | | 45 (11.25) |
| 11. You think that if the risk of contracting dengue is low, vaccination is unnecessary. | 64  (16) | 147 (36.75) | 93 (23.25) | 83 (20.75) | | 13 (3.25) |
| 12. You think that if there is no dengue outbreak nearby, vaccination is unnecessary. | 79 (19.75) | 154 (38.5) | 76  (19) | 82 (20.5) | | 9 (2.25) |
| **Total score (possible range = 0-48)**  **Means (SD)**  **Range** | 25.75 (8.5)  4-48 | | | | | |
| **Health Information Sources (multiple answers allowed)** | | | | | **n(%)** | |
| Advice from medical professionals | | | | | 344 (86) | |
| Conversations with others, such as other caregivers, family members, or friends | | | | | 215 (53.75) | |
| Internet search engines (e.g., Google, Yahoo!) | | | | | 298 (74.5) | |
| Social media (e.g., Facebook, X [formerly Twitter], TikTok, Instagram, YouTube) | | | | | 280 (70) | |
| Messaging apps (e.g., Line, WhatsApp, Messenger) | | | | | 149 (37.25) | |
| Government or public health organization websites (e.g., Ministry of Public Health, Department of Disease Control, hospital websites) | | | | | 213 (53.25) | |
| Health information websites (e.g., haamor.com, pobpad.com) | | | | | 181 (45.25) | |
| Websites of healthcare products and brands (e.g., vaccine brands) | | | | | 88 (22) | |
| Product or brand blogs/vlogs | | | | | 54 (13.5) | |
| Medical science journals (e.g., PubMed, New England Journal of Medicine) | | | | | 86 (21.5) | |
| Q&A forums (e.g., Pantip, Quora, Reddit) | | | | | 58 (14.5) | |
| Newspapers, magazines | | | | | 86 (21.5) | |
| Television | | | | | 195 (48.75) | |
| Radio | | | | | 65 (16.25) | |
| **Dengue vaccine acceptance** | | | | | **n(%)** | |
| Have you ever received the dengue vaccine?  - Never  - Yes: (n=15)  Have not completed/waiting for the next appointment  Have completed (n=8)  2 shots  3 shots | | | | | 386 (96.5)  15 (3.75)  7 (46.67)  8 (53.33)  6 (75)  2 (25) | |
| Have any of your children received the dengue vaccine? (n,parents = 400)  - Never  - Yes: (n,children = 47)  Have not completed/waiting for the next appointment  Stopped the DF vaccine  Have completed (n,children = 21)  2 shots  3 shots | | | | | 361 (90.25)  39 (9.75)  23 (48.94)  3 (6.38)  21 (44.68)  19 (90.48)  2 (9.52) | |
| If your child has never received the dengue vaccine, do you plan to have them vaccinated in the future? (n=361)  - Yes  - No | | | | | 215 (59.56)  146 (40.44) | |
| At least one of their kids has received the vaccine or plan to take them to get vaccinated | | | | | 254 (63.5) | |

^a^SD= strongly disagree, D=disagree, NO=no opinion/do not know, A=agree, SA=strongly agree
